# Sedentary behaviour and physical activity are associated with biomarkers of endothelial dysfunction and low-grade inflammation—relevance for (pre)diabetes: The Maastricht Study

**DOI:** 10.1007/s00125-022-05651-3

**Published:** 2022-02-04

**Authors:** Evelien J. Vandercappellen, Annemarie Koster, Hans H. C. M. Savelberg, Simone J. P. M. Eussen, Pieter C. Dagnelie, Nicolaas C. Schaper, Miranda T. Schram, Carla J. H. van der Kallen, Marleen M. J. van Greevenbroek, Anke Wesselius, Casper G. Schalkwijk, Abraham A. Kroon, Ronald M. A. Henry, Coen D. A. Stehouwer

**Affiliations:** 1 Department of Internal Medicine, Maastricht University Medical Center+, Maastricht, the Netherlands; 2 CARIM School for Cardiovascular Diseases, Maastricht University, Maastricht, the Netherlands; 3 CAPHRI Care and Public Health Research Institute, Maastricht University, Maastricht, the Netherlands; 4 Department of Social Medicine, Maastricht University, Maastricht, the Netherlands; 5 Department of Nutrition and Movement Science, Maastricht University, Maastricht, the Netherlands; 6 NUTRIM School for Nutrition and Translational Research in Metabolism, Maastricht University, Maastricht, the Netherlands; 7 Department of Epidemiology, Maastricht University, Maastricht, the Netherlands; 8 MHeNS School for Mental Health and Neuroscience, Maastricht University, Maastricht, the Netherlands; 9 Heart and Vascular Center, Maastricht University Medical Center+, Maastricht, the Netherlands; 10 Department of Complex Genetics and Epidemiology, Maastricht University, Maastricht, the Netherlands

**Keywords:** Cohort study, Diabetes mellitus type 2, Endothelial dysfunction, Low-grade inflammation, Physical activity, Sedentary behaviour

## Abstract

**Aims/hypothesis:**

Biomarkers of endothelial dysfunction and low-grade inflammation are important in the pathogenesis of CVD and can potentially be modified by physical activity and sedentary behaviour. Effects of physical activity on biomarkers of endothelial dysfunction may be especially prominent in type 2 diabetes.

**Methods:**

In the population-based Maastricht Study (*n* = 2363, 51.5% male, 28.3% type 2 diabetes, 15.1% prediabetes [defined as impaired glucose tolerance and impaired fasting glucose]), we determined biomarkers of endothelial dysfunction and low-grade inflammation, and combined *z* scores were calculated. Physical activity and sedentary behaviour were measured by activPAL. Linear regression analyses were used with adjustment for demographic, lifestyle and cardiovascular risk factors.

**Results:**

The association between total, light, moderate-to-vigorous and vigorous intensity physical activity and sedentary time on the one hand and biomarkers of endothelial dysfunction on the other were generally significant and were consistently stronger in prediabetes and type 2 diabetes as compared with normal glucose metabolism status (*p* for interaction <0.05). Associations between physical activity and sedentary behaviour on the one hand and low-grade inflammation on the other were also significant and were similar in individuals with and without (pre)diabetes (*p* for interaction >0.05).

**Conclusions/interpretation:**

Physical activity and sedentary behaviour are associated with biomarkers of endothelial dysfunction and low-grade inflammation. For biomarkers of endothelial dysfunction, associations between physical activity and sedentary behaviour were consistently stronger in (pre)diabetes than in normal glucose metabolism. Whether increasing physical activity or decreasing sedentary time can positively influence biomarkers of endothelial dysfunction in individuals with prediabetes and type 2 diabetes requires further study.

**Graphical abstract:**

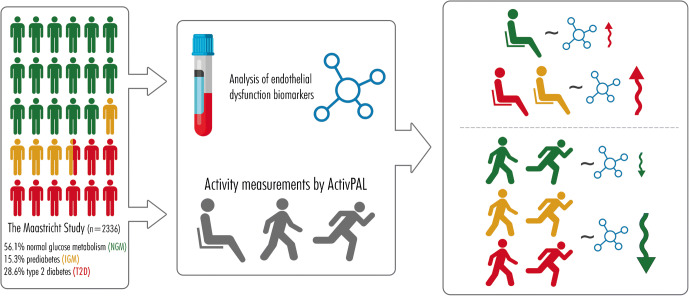

**Supplementary Information:**

The online version of this article (10.1007/s00125-022-05651-3) contains peer-reviewed but unedited supplementary material.

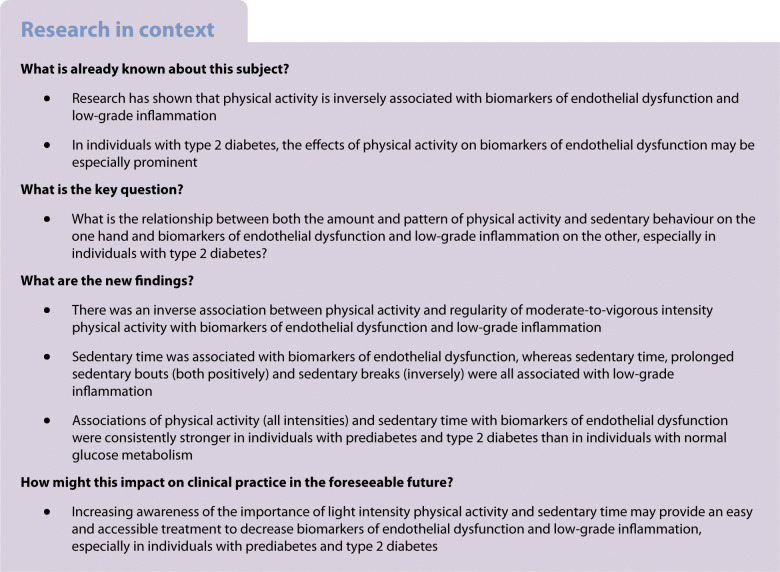



## Introduction

Biomarkers of endothelial dysfunction and low-grade inflammation play an important role in the pathogenesis of CVD [1–6]. Lifestyle factors, notably physical activity and diet, are thought to be important modifiable risk factors for the development of endothelial dysfunction and low-grade inflammation [7–9]. Physical activity in particular has been shown to be inversely related to biomarkers of endothelial dysfunction and low-grade inflammation [7, 9–17], possibly because physical activity increases blood flow and shear stress, which improves NO bioavailability [7] and because physical activity reduces visceral adipose tissue, which in turn reduces low-grade inflammation [8, 18]. The effects of physical activity on biomarkers of endothelial dysfunction may be especially prominent in individuals with type 2 diabetes mellitus. Hyperglycaemia impairs microvascular endothelial function and, specifically, reduces NO availability [19, 20]. Additionally, microvascular endothelial dysfunction (of which these biomarkers are a proxy) is thought to worsen hyperglycaemia through impairment of both insulin-induced glucose uptake and insulin secretion, thus establishing a vicious cycle of biomarkers of endothelial dysfunction and hyperglycaemia [20].

Physical activity is a complex behaviour in which several dimensions can be recognised, such as amount, intensity (light, moderate and vigorous) and weekly pattern. In addition, sedentary behaviour should be taken into account, and may be related to adverse health outcomes independently of moderate-to-vigorous intensity physical activity. Such distinctions may be relevant to provide public health guidelines that are more detailed than weekly amount of moderate-to-vigorous intensity physical activity [21].

In view of the above, in a large population-based cohort with an oversampling of type 2 diabetes, we investigated the relationship between both the amount and pattern of physical activity and sedentary behaviour on the one hand and biomarkers of endothelial dysfunction and low-grade inflammation on the other. Specifically, we focused on the influence of prediabetes (defined as impaired glucose tolerance and impaired fasting glucose) and type 2 diabetes on these associations.

## Methods

### Study population

We used data from The Maastricht Study, an observational prospective population-based cohort study. The rationale and methodology have been described previously [22].

In brief, the study focuses on the aetiology, pathophysiology, complications and comorbidities of type 2 diabetes and is characterised by an extensive phenotyping approach. Eligible participants were individuals between 40 and 75 years of age and living in the southern part of the Netherlands. Participants were recruited through mass media campaigns and from the municipal registries and the regional Diabetes Patient Registry via mailings. Recruitment was stratified according to known type 2 diabetes status, with an oversampling of individuals with type 2 diabetes, for reasons of efficiency. This study included cross-sectional data from 3451 participants, who completed the baseline survey between November 2010 and September 2013. The examinations of each participant were performed within a time window of 3 months.

The study was approved by the institutional medical ethical committee (NL31329.068.10) and the Minister of Health, Welfare and Sport of the Netherlands (permit no. 131088-105234-PG). All participants gave written informed consent.

### Measurements

#### Markers of low-grade inflammation and biomarkers of endothelial dysfunction

Plasma biomarkers of low-grade inflammation (high-sensitivity C-reactive protein [hs-CRP], serum amyloid A [SAA], soluble intercellular adhesion molecule-1 [sICAM-1], IL-6, IL-8, TNF-α) and biomarkers of endothelial dysfunction (sICAM-1, soluble vascular adhesion molecule-1 [sVCAM-1], soluble E-selectin [sE-selectin]) of the first 866 individuals of The Maastricht Study were measured in EDTA plasma samples with commercially available 4-plex sandwich immunoassay kits (Meso Scale Discovery, Rockville, USA) as described previously [23]. From individual 867 onwards, plasma biomarkers were measured in EDTA plasma samples with renewed commercially available 4-plex sandwich immunoassay kits with different standards and antibodies because of a change in the set-up of the ELISA plates measurements by Meso Scale Discovery. For this technique in this study, the intra- and inter-assay per cent CVs were 5.4 and 5.4 for hs-CRP, 8.7 and 10.8 for SAA, 10.3 and 8.4 for sICAM-1, 13.2 and 11.9 for IL-6, 7.6 and 5.5 for IL-8, 4.3 and 6.2 for TNF-α, 5.0 and 4.7 for sVCAM-1, and 2.9 and 7.4 for sE-selectin, respectively.

Absolute values of plasma biomarkers differed between individuals measured with the initial and renewed 4-plex sandwich immunoassay kits. To realign absolute values measured with the initial 4-plex sandwich immunoassay to the renewed 4-plex sandwich immunoassay, realignment formulas were calculated with Deming regression analyses [24]. In order to do so, 419 of the initial 866 individuals whose biomarkers were measured with the initial 4-plex sandwich immunoassay were also measured with the renewed 4-plex sandwich immunoassay. The endothelial dysfunction von Willebrand factor (vWF) was quantified in citrate plasma using ELISA (Dako, Glostrup, Denmark). The intra- and inter-assay CVs were 3.0% and 4.3%, respectively.

#### Physical activity, activity patterns and sedentary behaviour

Physical activity levels were measured using the activPAL3 physical activity monitor (PAL Technologies, Glasgow, UK). The activPAL3 is a small (53 × 35 × 7 mm), lightweight (15 g) triaxial accelerometer that records movement in the vertical, anteroposterior and mediolateral axes, and also determines posture (sitting or lying, standing and stepping) based on acceleration information. During the first research visit, the device was attached directly to the skin on the front of the right thigh with transparent 3M Tegaderm tape, after the device had been waterproofed using a nitrile sleeve. Participants were asked to wear the accelerometer for 8 consecutive days, without removing it at any time. To avoid inaccurately identifying non-wear time, participants were asked not to replace the device once removed. Data were uploaded using the activPAL software and processed using customised software written in MATLAB R2018b (MathWorks, Natick, USA) [25]. Data from the first day were excluded from the analysis because participants performed physical function tests at the research centre after the device was attached. In addition, data from the final wear day providing ≤14 h of out-of-bed data were excluded from the analysis. Participants were included if they provided at least 1 valid day (≥10 h of out-of-bed data) and at least 6 valid days for the pattern.

We calculated the amount of time per day spent in light intensity physical activity (defined as standing and <100 steps/min), moderate-to-vigorous intensity physical activity (defined as ≥100 steps/min), and vigorous intensity physical activity (defined as ≥130 steps/min) [26]. Total physical activity per day was defined as mean time spent stepping during out-of-bed time. Weekly activity pattern categories based on moderate-to-vigorous intensity physical activity were defined as: insufficiently active, <150 min moderate-to-vigorous intensity physical activity/week; and sufficiently active, ≥150 min moderate-to-vigorous intensity physical activity/week. The sufficiently active category was further subdivided into ‘weekend warrior’ and regularly active. In accordance with previous research, weekend warriors were defined as participants who did ≥50% of the weekly moderate-to-vigorous intensity physical activity on only 1 or 2 days [27]. Regularly active were participants who did their moderate-to-vigorous intensity physical activity in ≥3 days. Thus, we defined three groups: (1) insufficiently active (0–150 min moderate-to-vigorous intensity physical activity/week); (2) weekend warrior (≥150 min moderate-to-vigorous intensity physical activity/week with more than 50% of the moderate-to-vigorous intensity physical activity in 1 or 2 days); and (3) regularly active (≥150 min moderate-to-vigorous intensity physical activity/week in ≥3 days). Also, we assessed the variation of moderate-to-vigorous intensity physical activity/week per individual as a continuous variable by calculating the CV (CV=SD/mean, for each individual).

The total amount of sedentary time was based on the sedentary posture (sitting or lying), and calculated as the mean time spent per day in a sedentary position during out-of-bed time. The method used to determine out-of-bed time has been described elsewhere [25]. In brief, an automated algorithm identified out-of-bed and in-bed times on an individual level on multiple days, i.e. different out-of-bed and in-bed times for each day for each participant. The algorithm is based on the number and duration of sedentary periods to identify in-bed times, and on the number and duration of active periods (standing or stepping) to identify out-of-bed times. The algorithm showed high accuracy in determining out-of-bed time compared with self-report, as the intra-class *r* was 0.79 (*p*<0.001) and the mean difference in out-of-bed time between both methods was 0.02 h (1.2 min), with limits of agreement of −1.1 to 1.2 h.

The number of sedentary breaks during out-of-bed time was determined as each transition from a sitting or lying position to standing or stepping, and the mean number of breaks per day was calculated. Sedentary time accumulated in a consecutive period ≥30 min was defined as a prolonged sedentary bout, and the mean number of prolonged sedentary bouts during out-of-bed time per day was calculated.

#### Covariates

Covariates which were extracted from questionnaires included sex, age, level of education, smoking status, energy intake, Dutch healthy diet index, mobility limitation and history of CVD. Smoking status was categorised into never, former, and current smoker. Level of education was assessed by questionnaire and categorised into low (no education, primary education and lower vocational education); medium (general secondary education, general vocational education, and higher secondary and pre-university education); and high (higher vocational education and university). Energy intake and dietary habits were obtained from a validated food frequency questionnaire [28] and calculated as the mean energy intake (kcal) per day and adherence to Dutch healthy diet index [29]. Mobility limitation was obtained from the 36-Item Short Form Health Survey questionnaire and was defined as having difficulty walking 500 m or climbing a flight of stairs. Prevalent CVD was defined as a self-reported history of myocardial infarction, ischaemic or haemorrhagic stroke, or percutaneous artery angioplasty of, or vascular surgery on, the coronary, abdominal, peripheral or carotid arteries. The use of lipid-modifying, antihypertensive and glucose-lowering medication was assessed during a medication interview [22]. Office systolic BP, ambulatory 24 h BP, BMI, waist circumference, HbA_1c_, fasting plasma glucose (FPG), 2 h plasma glucose (2hPG), triacylglycerol and total cholesterol/HDL-cholesterol ratio were determined as described elsewhere [22]. Glucose metabolism status was assessed by medication use and 2 h OGTT and classified into normal glucose metabolism, prediabetes (defined as impaired glucose tolerance [FPG <7.0 mmol/l and 2hPG between ≥7.8 and <11.1 mmol/l] and impaired fasting glucose [FPG between 6.1 and 6.9 mmol/l and 2hPG <7.8 mmol/l]) and type 2 diabetes according to WHO 2006 criteria [30]. More extensive information of the covariates has been described previously [22].

### Statistical analyses

All data were analysed using IBM SPSS software version 25.0 for Windows (IBM, Armonk, NY). Characteristics of the total study population and according to moderate-to-vigorous intensity physical activity patterns were summarised as mean (SD) or as percentages or as median (IQR) (in case of a skewed distribution). To increase statistical efficiency and reduce multiple testing, an endothelial dysfunction *z* score and low-grade inflammation *z* score was calculated.

For endothelial dysfunction we combined: sICAM-1, sVCAM-1, sE-selectin and vWF.

For low-grade inflammation we combined: hs-CRP, IL-6, IL-8, TNF-α, SAA and sICAM-1.

First, we calculated the *z* scores for all individual biomarkers [(individual value minus whole study population mean value)/whole study population SD, thus resulting in a standardised variable ranging from approximately −2.5 to +2.5 SD with a mean of 0].

All the individual *z* scores from biomarkers share the same unit. They were averaged, resulting in one single endothelial dysfunction or low-grade inflammation score, which was subsequently standardised. sICAM-1 was included in both biomarker scores as it is expressed by both monocytes and the endothelium [2, 31–33]. A higher score indicates more biomarkers of endothelial dysfunction or more low-grade inflammation.

Associations between physical activity (total, light intensity, moderate-to-vigorous intensity combined and vigorous intensity separately), sedentary time/breaks, prolonged sedentary bouts and physical activity pattern (independent variables) and biomarkers of endothelial dysfunction and low-grade inflammation scores (dependent variables) were examined with the use of multivariable linear regression models. Models 1 and 2 are about potential confounding, and model 3 is about potential confounding/mediation. Model 1 was adjusted for age, sex and glucose metabolism status; model 2 was additionally adjusted for smoking status, Dutch healthy diet index and level of education; model 3 was additionally adjusted for history of CVD, BMI, office systolic BP, mobility limitations (yes/no), total cholesterol/HDL-cholesterol ratio, triacylglycerol, lipid-modifying medication and antihypertensive medication. The time out-of-bed can be defined as sedentary time, light intensity and moderate-to-vigorous intensity physical activity. Therefore, it may be that sedentary time is a confounder/mediator for moderate-to-vigorous intensity and vigorous intensity physical activity in the above analyses (model 4a). Sedentary behaviour and light intensity physical activity were further adjusted for moderate-to-vigorous intensity physical activity, as a possible confounder/mediator, in the above analyses (model 4a). Finally, we performed interaction analyses for sex, age and glucose metabolism status in model 3.

#### Additional analyses

Next, we conducted several sensitivity analyses: we excluded individuals with hs-CRP >10 mg/l; we replaced BMI with waist circumference; we excluded individuals with <6 days of activPAL; and we replaced office systolic BP with 24 h BP. Finally, because biomarkers of endothelial dysfunction and low-grade inflammation are thought to be related processes, they may act as mediators in the above analyses. To test whether this was the case, we adjusted associations with biomarkers of endothelial dysfunction for low-grade inflammation, and vice versa (model 4b). Finally, for the interpretation of the interaction analyses with glucose metabolism status, we performed the interaction analyses described above for glucose metabolism status with HbA_1c_ and FPG and 2hPG values of the OGTT.

For all analyses, a *p* value <0.05 was considered statistically significant.

## Results

The study population for all analyses, except those for activity patterns and stratified by glucose metabolism status, consisted of 2363 participants. We excluded 810 with no accelerometer measurements, 38 with missing biomarkers of endothelial dysfunction or low-grade inflammation and 240 who had other missing data (confounders) from the overall study population (*n* = 3451). Activity pattern analyses were done in 2015 participants, as 348 participants were additionally excluded because of accelerometer measurements of less than 6 days. For the analyses stratified by glucose metabolism status, we excluded other types of diabetes. In total we performed the stratified analysis with 2336 participants. We compared the baseline characteristics of the included and excluded populations and found that the characteristics were similar (electronic supplementary material [ESM] Table 1).

Table 1 shows the characteristics of the total study population and according to the pattern of physical activity. The insufficiently actives, as compared with the more active participants, were older, were more often current smokers, more often had mobility limitations, more often had type 2 diabetes, more often used medication, and had higher levels of biomarkers of endothelial dysfunction and low-grade inflammation.

**Table 1:** Descriptive characteristics of the study population according to physical activity pattern

		Physical activity pattern (*n*=2015)		
Characteristic	Total population (*n*=2363)	Insufficiently active (*n*=173)	Weekend warrior (*n*=494)	Regularly active (*n*=1348)
Age (years)	61.0 (55.0–66.0)	64.0 (59.0–70.0)	63.0 (56.0–67.0)	60.0 (54.0–66.0)
Sex (% male)	51.5	76.3	57.3	44.6
Education level (%)				
Low	33.5	50.9	33.0	31.1
Medium	28.1	28.9	24.3	28.6
High	38.4	20.2	42.7	40.4
Smoking status (%)				
Current	12.5	24.9	10.1	10.3
Former	52.2	53.2	52.4	52.0
Never	35.3	22.0	37.4	37.7
Alcohol consumption (%)				
None	17.6	28.9	14.0	16.9
Low	57.0	54.9	56.7	57.3
High	25.4	16.2	29.4	25.8
Mobility limitations (%)				
Yes	20.3	46.8	17.8	17.4
No	79.7	53.2	82.2	82.6
BMI (kg/m^2^)	27.02 (4.54)	30.11 (5.51)	26.82 (4.09)	26.42 (4.11)
History of CVD (%)	16.6	30.1	16.4	15.0
Diabetes status (%)				
Normal	55.5	23.1	55.7	61.6
Impaired	15.1	10.4	16.8	14.8
Type 2 diabetes	28.3	64.7	26.7	22.4
Other type of diabetes	1.1	1.7	0.8	1.2
Antihypertensive medication use (%)	41.4	69.4	42.7	37.8
Lipid-modifying medication use (%)	37.7	64.2	37.9	32.9
Glucose-lowering medication use (%)	23.7	58.4	21.7	18.4
Total cholesterol/HDL-cholesterol ratio	3.38 (2.76–4.19)	3.64 (3.00–4.60)	3.45 (2.82–4.29)	3.28 (2.70–4.06)
Triacylglycerol (mmol/l)	1.22 (0.89–1.72)	1.54 (1.12–2.14)	1.25 (0.90–1.75)	1.16 (0.86–1.61)
Dutch healthy diet index	83.73 (14.67)	78.19 (14.80)	84.39 (13.63)	85.08 (14.81)
Valid days (*n*)	7.0 (6.00–7.00)	7.00 (6.00–7.00)	7.0 (6.00–7.00)	7.0 (6.00–7.00)
Sedentary time (h/day)	9.42 (1.67)	10.96 (1.51)	9.66 (1.44)	9.09 (1.55)
Total physical activity (h/day)	1.96 (1.49–2.44)	1.01 (0.81–1.21)	1.85 (1.50–2.25)	2.09 (1.68–2.55)
Light intensity physical activity (h/day)	5.41 (1.52)	4.12 (1.39)	5.07 (1.30)	5.69 (1.43)
Moderate-to-vigorous intensity physical activity (h/day)	0.85 (0.58–1.15)	0.27 (0.19–0.31)	0.84 (0.64–1.10)	0.94 (0.68–1.22)
Vigorous intensity physical activity (h/day)	0.09 (0.04–0.19)	0.02 (0.01–0.03)	0.09 (0.05–0.18)	0.10 (0.06–0.21)
Sedentary breaks (*n*/day)	37.45 (8.58)	33.17 (8.21)	35.50 (7.63)	38.51 (8.25)
Prolonged sedentary bouts (*n*/day)	4.86 (3.71–5.86)	6.29 (5.17–7.17)	5.29 (4.33–6.14)	4.46 (3.50–5.50)
sICAM-1 (ng/ml)	355.62 (98.26)	402.95 (144.46)	353.75 (94.21)	348.68 (85.17)
sVCAM-1 (ng/ml)	428.50 (100.61)	471.35 (135.04)	432.68 (99.96)	421.59 (91.75)
sE-selectin (ng/ml)	117.81 (66.46)	154.91 (112.77)	114.12 (58.05)	112.39 (59.95)
von Willebrand factor (%)	132.20 (47.84)	154.03 (52.48)	134.60 (51.34)	128.32 (44.64)
Plasma hs-CRP (mg/l)	1.22 (0.61–2.72)	1.99 (1.01–4.04)	1.21 (0.64–2.84)	1.12 (0.57–2.45)
Plasma SAA (μg/ml)	3.36 (2.09–5.45)	3.48 (2.10–6.89)	3.30 (2.14–5.31)	3.36 (2.04–5.36)
Plasma IL-6 (pg/ml)	0.58 (0.39–0.90)	0.89 (0.65–1.42)	0.57 (0.42–0.87)	0.55 (0.37–0.84)
Plasma IL-8 (pg/ml)	4.11 (3.25–5.29)	4.83 (3.89–6.42)	4.23 (3.34–5.27)	3.97 (3.19–5.13)
Plasma TNF-α (pg/ml)	2.20 (1.89–2.56)	2.50 (2.19–3.00)	2.18 (1.86–2.55)	2.16 (1.87–2.51)

Values are means (SD) or median (Q1–Q3), unless stated otherwise

After adjustment for demographic, lifestyle and cardiovascular risk factors, more physical activity (total and all intensities of physical activity) and more sedentary breaks were inversely associated with biomarkers of endothelial dysfunction (standardised β [95% CI], total: −0.11 [−0.15, −0.07]; light: −0.05 [−0.09, −0.02]; moderate-to-vigorous: −0.09 [−0.13, −0.05]; vigorous: −0.04 [−0.08, −0.004]; sedentary breaks: −0.04 [−0.08, −0.002]) (Table 2, model 3). Additionally, more sedentary time and more prolonged sedentary bouts were positively associated with biomarkers of endothelial dysfunction (sedentary time: 0.08 [0.04, 0.12]; prolonged sedentary bouts: 0.06 [0.02, 0.10]) (Table 2, model 3).

**Table 2:** Associations of physical activity and sedentary behaviour with biomarkers of endothelial dysfunction score and low-grade inflammation score

Physical activity level	Biomarkers of endothelial dysfunction					Low-grade inflammation				
	Model 1 β (95% CI)	Model 2 β (95% CI)	Model 3 β (95% CI)	Model 4a β (95% CI)	Model 4b β (95% CI)	Model 1 β (95% CI)	Model 2 β (95% CI)	Model 3 β (95% CI)	Model 4a β (95% CI)	Model 4b β (95% CI)
Total physical activity (h/day)	−0.18 (−0.22, −0.14)***	−0.17 (−0.21, −0.13)***	−0.11 (−0.15, −0.07)***	–	−0.04 (−0.07, −0.01)*	−0.22 (−0.26, −0.18)***	−0.20 (−0.24, −0.16)***	−0.11 (−0.15, −0.08)***	–	−0.06 (−0.09, −0.03)***
Light intensity physical activity (h/day)	−0.10 (−0.14, −0.06)***	−0.10 (−0.14, −0.06)***	−0.05 (−0.09, −0.02)**	−0.04 (−0.08, 0.001)	−0.01 (−0.04, 0.02)	−0.14 (−0.18, −0.10)***	−0.13 (−0.17, −0.09)***	−0.07(−0.11, −0.04)***	−0.05 (−0.09, −0.02)**	−0.04 (−0.07, −0.01)**
Moderate-to-vigorous intensity physical activity (h/day)	−0.16 (−0.20, −0.13)***	−0.15 (−0.19, −0.11)***	−0.09 (−0.13, −0.05)***	−0.08 (−0.12, −0.04)**	−0.02 (−0.06, 0.01)	−0.22 (−0.26, −0.18)***	−0.19 (−0.23, −0.15)***	−0.11 (−0.15, −0.07)***	−0.09 (−0.13, −0.05)***	−0.06 (−0.10, −0.03)***
Vigorous intensity physical activity (h/day)	−0.10 (−0.14, −0.06)***	−0.09 (−0.13, −0.05)***	−0.04 (−0.08, −0.004)*	–0.03 (−0.07, 0.01)	−0.004 (−0.04, 0.03)	−0.15 (−0.19, −0.11)***	−0.13 (−0.17, −0.09)***	−0.07 (−0.10, −0.03)***	−0.06 (−0.09, −0.02)**	−0.04 (−0.07, −0.01)**
Sedentary time (h/day)	0.13 (0.09, 0.17)***	0.13 (0.09, 0.17)***	0.08 (0.04, 0.12)***	0.06 (0.02, 0.10)**	0.03 (−0.001, 0.07)	0.16 (0.12, 0.20)***	0.15 (0.11, 0.19)***	0.08 (0.05, 0.12)***	0.05 (0.01, 0.09)*	0.04 (0.01, 0.07)*
Sedentary breaks (*n*/day)	−0.08 (−0.12, −0.04)***	−0.08 (−0.12, −0.04)***	−0.04 (−0.08, −0.002)*	−0.03 (−0.07, 0.01)	−0.01 (−0.04, 0.02)	−0.11 (−0.15, −0.07)***	−0.10 (−0.14, −0.07)***	−0.05 (−0.09, −0.02)**	−0.04 (−0.08, −0.004)*	−0.03 (−0.06, −0.002)*
Prolonged sedentary bouts (*n*/day)	0.11 (0.07, 0.15)***	0.12 (0.08, 0.16)***	0.06 (0.02, 0.10)**	0.04 (−0.003, 0.08)	0.02 (−0.02, 0.05)	0.15 (0.11, 0.19)***	0.15 (0.11, 0.19)***	0.08 (0.04, 0.11)***	0.05 (0.01, 0.09)*	0.05 (0.01, 0.08)**

Regression coefficients (β) represents the increase/decrease in biomarkers of endothelial dysfunction/low-grade inflammation for every SD physical activity/sedentary behaviour

1 SD total physical activity is equivalent to 0.69 h/day; 1 SD light intensity physical activity is equivalent to 1.52 h/day; 1 SD moderate-to-vigorous intensity physical activity is equivalent to 0.45 h/day; 1 SD vigorous intensity physical activity is equivalent to 0.17 h/day; 1 SD sedentary time is equivalent to 1.67 h/day; 1 SD sedentary breaks is equivalent to 8.58 breaks/day; 1 SD prolonged sedentary bouts is equivalent to 1.57 bouts/day

Model 1 was adjusted for age, sex and glucose metabolism status; model 2 was additionally adjusted for smoking, Dutch healthy diet index and level of education; model 3 was additionally adjusted for history of CVD, BMI, mobility limitation (yes/no), triacylglycerol, total cholesterol/HDL-cholesterol ratio, use of lipid-modifying medication, use of antihypertensive medication and office systolic BP

For the sedentary behaviour and light intensity physical activity, model 4a was additionally adjusted for moderate-to-vigorous intensity physical activity; for moderate-to-vigorous intensity physical activity and vigorous intensity physical activity, model 4a was additionally adjusted for sedentary time; model 4b was additionally adjusted for low-grade inflammation (in case of biomarkers of endothelial dysfunction) and biomarkers of endothelial dysfunction (in case of low-grade inflammation)

*p<0.05, **p<0.01, ***p<0.001

After adjustment for demographic, lifestyle and cardiovascular risk factors, more physical activity (total and all intensities of physical activity) and more sedentary breaks were inversely associated with the low-grade inflammation score (total: −0.11 [−0.15, −0.08]; light: −0.07 [−0.11, −0.04]; moderate-to-vigorous: −0.11 [−0.15, −0.07]; vigorous: −0.07 [−0.10, −0.03]; sedentary breaks: −0.05 [−0.09, −0.02]) (Table 2, model 3). Additionally, more sedentary time and more prolonged sedentary bouts were positively associated with the low-grade inflammation score (sedentary time: 0.08 [0.05, 0.12]; prolonged sedentary bouts: 0.08 [0.04, 0.11]) (all *p*<0.05; Table 2, model 3).

To investigate the mutual independence of the above associations, we adjusted light intensity physical activity and sedentary behaviour for moderate-to-vigorous intensity physical activity, and moderate-to-vigorous intensity and vigorous intensity physical activity for sedentary time. For biomarkers of endothelial dysfunction score, only moderate-to-vigorous intensity physical activity and sedentary time remained associated (Table 2, model 4a). For the low-grade inflammation score, associations remained similar for light intensity physical activity, moderate-to-vigorous intensity and vigorous intensity physical activity, sedentary time, sedentary breaks and prolonged sedentary bouts (Table 2, model 4a).

After adjustment for demographic, lifestyle and cardiovascular risk factors, regularly actives and weekend warriors, as compared with the insufficiently actives (as reference), had comparably lower biomarkers of endothelial dysfunction and low-grade inflammation scores (biomarkers of endothelial dysfunction: weekend warriors −0.28 [−0.44, −0.11]; regularly actives −0.32 [−0.47, −0.16], low-grade inflammation: weekend warriors −0.22 [−0.38, −0.07]; regularly actives −0.26 [−0.41, −0.11]) (Table 3, model 3). However, the CV of the weekly amount of moderate-to-vigorous intensity physical activity (regularity) was positively associated with biomarkers of endothelial dysfunction and low-grade inflammation scores (biomarkers of endothelial dysfunction: 0.04 [0.01, 0.07], low-grade inflammation: 0.05 [0.02, 0.08]) (Table 3, model 3).

**Table 3:** Associations of physical activity pattern and CV and biomarkers of endothelial dysfunction score and low-grade inflammation score

Physical activity pattern	Biomarkers of endothelial dysfunction			Low-grade inflammation		
	Model 1 β (95% CI)	Model 2 β (95% CI)	Model 3 β (95% CI)	Model 1 β (95% CI)	Model 2 β (95% CI)	Model 3 β (95% CI)
Insufficiently active	Ref	Ref	Ref	Ref	Ref	Ref
Weekend warrior	−0.45 (−0.61, −0.28)***	−0.41 (−0.58, −0.24)***	−0.28 (−0.44, −0.11)**	−0.48 (−0.64, −0.31)***	−0.39 (−0.56, −0.23)***	−0.22 (−0.38, −0.07)**
Regularly active	−0.51 (−0.66, −0.35)***	−0.47 (−0.63, −0.31)***	−0.32 (−0.47, −0.16)***	−0.54 (−0.69, −0.39)***	−0.46 (−0.61, −0.31)***	−0.26 (−0.41, −0.11)***
CV	0.08 (0.05, 0.11)***	0.07 (0.04, 0.10)***	0.04 (0.01, 0.07)*	0.11 (0.08, 0.14)***	0.09 (0.06, 0.12)***	0.05 (0.02, 0.08)**

Regression results are presented as unstandardised coefficients (β), with 95% CIs

Model 1 was adjusted for age, sex and glucose metabolism status; model 2 was additionally adjusted for smoking, Dutch healthy diet index and level of education; model 3 was additionally adjusted for history of CVD, BMI, office systolic BP, mobility limitation (yes/no), triacylglycerol, total cholesterol/HDL-cholesterol ratio, use of lipid-modifying medication and use of antihypertensive medication

*p<0.05, **p<0.01, ***p<0.001

Associations of physical activity and sedentary behaviour with individual biomarkers of endothelial dysfunction and low-grade inflammation are shown in ESM Tables 2–10. In general, associations were similar to those with biomarkers of endothelial dysfunction and low-grade inflammation scores.

Associations between light intensity physical activity and prolonged sedentary bouts on the one hand and low-grade inflammation (but not biomarkers of endothelial dysfunction) on the other were stronger in women than in men (*p* for interaction <0.05; ESM Table 11 shows stratified analyses). There was no interaction with age.

Associations between total physical activity, light intensity physical activity, moderate-to-vigorous intensity physical activity, vigorous intensity physical activity and sedentary time on the one hand and biomarkers of endothelial dysfunction (but not low-grade inflammation) on the other were in general consistently stronger in individuals with prediabetes and type 2 diabetes than in those without (*p* for interaction <0.05; standardised β [95% CI] total physical activity: −0.16 [−0.26, −0.05], −0.14 [−0.22, −0.06], −0.06 [−0.12, −0.01]; light intensity physical activity: −0.17 [−0.27, −0.06], −0.10 [−0.18, −0.03], 0.02 [−0.04, 0.07]; moderate-to-vigorous intensity physical activity: −0.11 [−0.21, −0.001], −0.10 [−0.18, −0.02], −0.08 [−0.13, −0.02]; vigorous intensity physical activity: −0.03 [−0.13, 0.08], −0.05 [−0.13, 0.02], −0.06 [−0.11, −0.002]; sedentary time: 0.21 [0.10, 0.31], 0.10 [0.02, 0.17], 0.02 [−0.04, 0.07]); Table 4 shows these stratified analyses. When we replaced glucose metabolism status with HbA_1c_, FPG or 2hPG in the interaction analyses with biomarkers of endothelial dysfunction, we observed significant interactions for total physical activity (FPG and 2hPG), light intensity physical activity (HbA_1c_, FPG and 2hPG), moderate-to-vigorous intensity physical activity (FPG and 2hPG) and sedentary time (FPG and 2hPG), but not for vigorous intensity physical activity (ESM Tables 12–14).

**Table 4:** Associations of physical activity and sedentary time with biomarkers of endothelial dysfunction, stratified by glucose metabolism status

Physical activity level	Biomarkers of endothelial dysfunction		
	Model 1 β (95% CI)	Model 2 β (95% CI)	Model 3 β (95% CI)
Total physical activity			
Normal glucose metabolism	−0.12 (−0.17, −0.06)***	−0.11 (−0.17, −0.06)***	−0.06 (−0.12, −0.01)*
Prediabetes	−0.20 (−0.30, −0.10)***	−0.21 (−0.31, −0.10)***	−0.16 (−0.26, −0.05)**
T2D	−0.27 (−0.34, −0.19)***	−0.25 (−0.32, −0.17)***	−0.14 (−0.22, −0.06)***
Light intensity physical activity			
Normal glucose metabolism	−0.02 (−0.08, 0.03)	−0.02 (−0.08, 0.03)	0.02 (−0.04, 0.07)
Prediabetes	−0.15 (−0.26, −0.04)**	−0.16 (−0.27, −0.05)**	−0.17 (−0.27, −0.06)**
T2D	−0.17 (−0.25, −0.10)***	−0.17 (−0.25, −0.10)***	−0.10 (−0.18, −0.03)**
Moderate-to-vigorous intensity physical activity			
Normal glucose metabolism	−0.13 (−0.18, −0.07)***	−0.12 (−0.17, −0.07)***	−0.08 (−0.13, −0.02)**
Prediabetes	−0.17 (−0.27, −0.07)**	−0.16 (−0.26, −0.06)**	−0.11 (−0.21, −0.001)*
T2D	−0.24 (−0.31, −0.16)***	−0.21 (−0.28, −0.13)***	−0.10 (−0.18, −0.02)*
Vigorous intensity physical activity			
Normal glucose metabolism	−0.11 (−0.16, −0.05)***	−0.10 (−0.16, −0.05)***	−0.06 (−0.11, −0.002)*
Prediabetes	−0.10 (−0.20, 0.01)	−0.09 (−0.19, 0.02)	−0.03 (−0.13, 0.08)
T2D	−0.15 (−0.22, −0.07)***	−0.12 (−0.20, −0.05)**	−0.05 (−0.13, 0.02)
Sedentary time			
Normal glucose metabolism	0.06 (0.001, 0.10)*	0.06 (0.003, 0.12)*	0.02 (−0.04, 0.07)
Prediabetes	0.20 (0.10, 0.31)***	0.22 (0.11, 0.33)***	0.21 (0.10, 0.31)***
T2D	0.18 (0.11, 0.26)***	0.18 (0.10, 0.25)***	0.10 (0.02, 0.17)*

Regression coefficients (β) represents the increase/decrease in biomarkers of endothelial dysfunction for every SD physical activity/sedentary behaviour

1 SD total physical activity is equivalent to 0.67 h/day, 1 SD light intensity physical activity is equivalent to 1.48 h/day, 1 SD moderate-to-vigorous intensity physical activity is equivalent to 0.43 h/day, 1 SD vigorous intensity physical activity is equivalent to 0.16 h/day, 1 SD sedentary time is equivalent to 1.60 h/day

Model 1 was adjusted for age and sex; model 2 was additionally adjusted for smoking, Dutch healthy diet index and level of education; model 3 was additionally adjusted for history of CVD, BMI, office systolic BP, mobility limitation (yes/no), triacylglycerol, total cholesterol/HDL-cholesterol ratio, use of lipid-modifying medication and use of antihypertensive medication

*p<0.05, **p<0.01, ***p<0.001

T2D, type 2 diabetes

### Additional analyses

To test the robustness of the above results, we did several sensitivity analyses. In general, results remained similar when we excluded participants with hs-CRP> 10 mg/l (ESM Tables 15 and 16); when we replaced BMI with waist circumference (ESM Tables 17 and 18); when we replaced office systolic BP with 24 h systolic BP (ESM Tables 19 and 20); and when we excluded participants with less than 6 days of activPAL (2015 individuals) (ESM Table 21).

Finally, from a pathophysiological point of view, biomarkers of endothelial dysfunction and low-grade inflammation are strongly interlinked [34, 35]. Indeed, when we adjusted associations with biomarkers of endothelial dysfunction as outcome for the low-grade inflammation score, or vice versa, associations of physical activity intensity or sedentary behaviour with these outcomes were consistently attenuated (Table 2, model 4b).

## Discussion

This cross-sectional population-based study on the associations between accelerometer-measured physical activity and sedentary behaviour on the one hand and biomarkers of endothelial dysfunction and low-grade inflammation on the other had three main findings. First, all intensities of physical activity and, in addition, regularity of moderate-to-vigorous intensity physical activity were inversely associated with biomarkers of endothelial dysfunction and low-grade inflammation. Second, and independently of moderate-to-vigorous intensity physical activity, sedentary time was associated with biomarkers of endothelial dysfunction, whereas sedentary time, prolonged sedentary bouts (both positively) and sedentary breaks (inversely) were all associated with low-grade inflammation. Third, associations between physical activity (all intensities) and sedentary time on the one hand and biomarkers of endothelial dysfunction on the other were consistently stronger in individuals with prediabetes and type 2 diabetes than in individuals with normal glucose metabolism.

All intensities of physical activity were inversely associated with biomarkers of endothelial dysfunction and low-grade inflammation. In general, our results are consistent with previous studies [12–15, 36–39]. As an important extension to previous publications, the present study had the advantage of a more extensive characterisation of both physical activity on the one hand and biomarkers of endothelial dysfunction and low-grade inflammation on the other. Importantly, we found that regularity (i.e. a lower CV, as a continuous variable) of moderate-to-vigorous intensity activity was associated with less biomarkers of endothelial dysfunction and low-grade inflammation. We attribute the observation that similar differences were not observed when regularly actives were compared with weekend warriors to the decrease in power associated with analyses with categorical variables.

Independently of moderate-to-vigorous intensity physical activity, sedentary time was associated with biomarkers of endothelial dysfunction, whereas sedentary time, prolonged sedentary bouts (both positively) and sedentary breaks (inversely) were all associated with low-grade inflammation. These results are generally consistent with those of previous studies [11, 40–43].

Physical activity is thought to ameliorate endothelial function through inducing shear stress, which in turn increases activity of endothelial oxide nitric synthase and enhances NO bioavailability [7]. Conversely, sedentary behaviour reduces shear stress, which decreases endothelial oxide nitric synthase and decreases bioavailability of NO [11]. However, the mechanisms linking physical activity and sedentary behaviour to low-grade inflammation are not completely understood. Possible explanations include a reduction in visceral adipose tissue, a reduction of toll-like receptors on immune cells, and changes in the number of (immune) cells [8, 18].

We found that associations between physical activity and biomarkers of endothelial dysfunction were consistently stronger in individuals with prediabetes and type 2 diabetes than in individuals with normal glucose metabolism. When analyses were repeated with measures of glucose (HbA_1c_, FPG or 2hPG), results were mostly but not entirely consistent, which may be due to the play of chance or reflect true differences between (pre)diabetes as a state and glucose measures that are not explained by the adjustments we made, and thus were not identified in this study.

Hyperglycaemia and impairment of microvascular endothelial function are thought to constitute a vicious cycle [20]. We speculate that physical activity, by improving microvascular endothelial function, interrupts this vicious cycle. In short-term studies, the effect on biomarkers of endothelial dysfunction of increasing physical activity has not been found to be larger in individuals with, than in those without, type 2 diabetes [39], but it must be recognised that activity measurements such as those obtained in the current study probably reflect long-term lifestyle habits. Thus, long-term exposures to greater physical activity may be needed to improve endothelial function in individuals with type 2 diabetes. This hypothesis requires further study in long-term intervention trials.

This study had several strengths: we studied a large population-based sample; we studied amount, intensity and pattern of physical activity as measured by accelerometry; we used multiple biomarkers of endothelial dysfunction and low-grade inflammation; and we took extensive measurements of possible confounders, which makes residual confounding less likely. The study also had several limitations. Because of the cross-sectional design, causal inference should be made with great caution, although our results are physiologically plausible. Earlier research showed that poorer health is associated with less physical activity and more sedentary behaviour [44–46]. Next, the use of composite biomarker scores assumes that all biomarkers are equally important in pathophysiological terms. Whether this assumption holds is not completely clear, but all biomarkers used have been shown to be prognostically linked to CVD [3–5], and associations were homogeneous across biomarkers. Furthermore, we have only used the activPAL, which unfortunately does not allow us to distinguish the specific type of activities (e.g. endurance exercise vs strength exercise and subtypes of exercise, such as walking, running, cycling, swimming). All activities are captured as activity by the activPAL, and we were further able to classify activities into light, moderate and vigorous intensity physical activity. Finally, the population consisted only of White participants, which limits generalisability of our findings.

In conclusion, our study shows that, independently of demographic, lifestyle and cardiovascular risk factors, different intensities of physical activity are inversely associated with biomarkers of endothelial dysfunction and low-grade inflammation. Additionally, greater regularity of moderate-to-vigorous intensity physical activity is advantageous with regard to biomarkers of endothelial dysfunction and low-grade inflammation. Sedentary time was directly associated with biomarkers of endothelial dysfunction, and sedentary time, prolonged sedentary bouts (positively) and sedentary breaks (inversely) were associated with low-grade inflammation, and this was independent of moderate-to-vigorous intensity physical activity. Importantly, associations of physical activity and biomarkers of endothelial dysfunction were consistently stronger in prediabetes and type 2 diabetes. Taken together, these results support increasing physical activity and limiting sedentary behaviour as a means to prevent or ameliorate biomarkers of endothelial dysfunction and low-grade inflammation, especially in individuals with prediabetes and type 2 diabetes.

## Supplementary information

ESM 1 (PDF 367 kb)

## Data Availability

The datasets generated during and/or analysed during the current study are not publicly available but are available from the corresponding author on reasonable request.

## Abbreviations

2hPG: 2 h plasma glucose
FPG: Fasting plasma glucose
hs-CRP: High-sensitivity C-reactive protein
SAA: Serum amyloid A
sE-selectin: Soluble E-selectin
sICAM-1: Soluble intercellular adhesion molecule-1
sVCAM-1: Soluble vascular adhesion molecule-1
vWF: von Willebrand factor
