# Supplementary material for: Sedentary behaviour and physical activity are associated with biomarkers of endothelial dysfunction and low-grade inflammation—relevance for (pre)diabetes: The Maastricht Study
Source: Diabetologia. 2022 Feb 4;65(5):777–89. doi: 10.1007/s00125-022-05651-3 (PMC8960649; doi:10.1007/s00125-022-05651-3)
Supplement: Supplementary file 1 — (PDF 367 kb) [file 125_2022_5651_MOESM1_ESM.pdf]

ESM table 1: Descriptive characteristics of the in- and exclusion study population

Characteristics	Inclusion (n=2363)	Exclusion (n=1088)
Age (years)	61.0 [55.0;66.0]	60.0 [53.0;66.0]
Sex (% male)	51.5	51.3
Education level (%)		
• Low	33.5	33.9
• Medium	28.1	28.6
• High	38.4	37.5
Smoking status (%)		
• Current	12.5	16.9
• Former	52.2	50.6
• Never	35.3	32.6
Alcohol consumption (%)		
• None	17.6	21.3
• Low	57.0	51.9
• High	25.4	26.8
Mobility limitations (%)		
• Yes	20.3	25.6
• Non	79.7	74.4
BMI (kg/m^2)	27.02 (4.54)	27.23 (4.60)
History of CVD (%)	16.6	17.2
Diabetes status (%)		
• Normal	55.5	56.3

• Impaired<br>• Type 2 diabetes<br>• Other type of diabetes	15.1<br>28.3<br>1.1	14.2<br>28.1<br>1.3
Antihypertensive medication use (%)	41.4	38.0
Lipid-modifying medication use (%)	37.7	33.8
Glucose-lowering medication use (%)	23.7	22.8
Total cholesterol-to-HDL cholesterol ratio	3.38 [2.76;4.19]	3.67 [3.00;4.54]
Triglycerides (mmol/L)	1.22 [0.89;1.72]	1.19 [0.86;1.73]
Dutch healthy diet index	83.73 (14.67)	81.95 (14.78)
Valid days (n)	7.0 [6.00;7.00]	6.00 [5.00;7.00]
Sedentary time (h/day)	9.42 (1.67)	9.36 (1.82)
Total physical activity (h/day)	1.96 [1.49;2.44]	1.91 [1.41;2.40]
Light intensity physical activity (h/day)	5.41 (1.52)	5.42 (1.65)
Moderate-to-vigorous intensity physical activity (h/day)	0.85 [0.58;1.15]	0.77 [0.50;1.06]
Vigorous intensity physical activity (h/day)	0.09 [0.04;0.19]	0.07 [0.03;0.17]
Sedentary breaks (n/day)	37.45 (8.58)	38.21 (8.95)
Prolonged sedentary bouts (n/day)	4.86 [3.71;5.86]	4.69 [3.67;5.71]
Soluble ICAM-1 (ng/ml)	355.62 (98.26)	353.56 (103.12)
Soluble VCAM-1 (ng/ml)	428.50 (100.61)	428 (104.10)
Soluble E-selectin (ng/ml)	117.81 (66.46)	120.14 (62.10)
Von Willebrand Factor (%)	132.20 (47.84)	134.48 (49.91)
Plasma CRP (µg/ml)	1.22 [0.61;2.72]	1.33 [0.64;2.97]
Plasma SAA (µg/ml)	3.36 [2.09;5.45]	3.08 [1.94;5.47]

Plasma IL-6 (pg/ml)	0.58 [0.39;0.90]	0.60 [0.39;0.92]
Plasma IL-8 (pg/ml))	4.11 [3.25;5.29]	4.20 [3.36;5.44]
Plasma TNFa (pg/ml)	2.20 [1.89;2.56]	2.19 [1.88;2.56]

Values are means (SD) or median [Q1-Q3], unless stated otherwise.

ESM table 2: Associations of physical activity and sedentary behavior with sICAM-1.

	sICAM-1		
	Model 1<br>B (95% CI)	Model 2<br>B (95% CI)	Model 3<br>B (95% CI)
Total physical activity (h/day)	-0.14 (-0.19;-0.10)***	-0.12 (-0.16;-0.08)***	-0.07 (-0.11;-0.02)**
Light intensity physical activity (h/day)	-0.08 (-0.12;-0.04)***	-0.07 (-0.11;-0.03)**	-0.03 (-0.07;0.01)
Moderate-to-vigorous intensity physical activity (h/day)	-0.14 (-0.18;-0.10)***	-0.11 (-0.15;-0.07)***	-0.06 (-0.10;-0.02)**
Vigorous intensity physical activity (h/day)	-0.09 (-0.13;-0.05)***	-0.08 (-0.12;-0.04)***	-0.03 (-0.07;0.01)
Sedentary time (h/day)	0.11 (0.07;0.15)***	0.10 (0.06;0.14)***	0.06 (0.01;0.10)*
Sedentary breaks (n/day)	-0.04 (-0.08;0.000)	-0.04 (-0.08;0.001)	-0.01 (-0.05;0.03)
Prolonged sedentary bouts (n/day)	0.07 (0.03;0.12)***	0.07 (0.03;0.11)***	0.03 (-0.01;0.07)

Regression coefficients (β) represents the increase/decrease in sICAM-1 behavior for every standard deviation (SD) physical activity/sedentary behavior. 1 SD total physical activity is equivalent to 0.69 h/day, 1 SD light intensity physical activity is equivalent to 1.52 h/day, 1 SD moderate-to-vigorous intensity physical activity is equivalent to 0.45 h/day, 1 SD vigorous intensity physical activity is equivalent to 0.17 h/day, 1 SD sedentary time is equivalent to 1.67 h/day, 1 SD sedentary breaks is equivalent to 8.58 breaks/day, 1 SD

prolonged sedentary bouts is equivalent to 1.57 bouts/day. Model 1 was adjusted for age, sex and glucose metabolism status; model 2 was additionally adjusted for smoking, Dutch healthy diet index and level of education; model 3 was additionally adjusted for history of CVD, BMI, mobility limitation (yes/no), triacylglycerol, total cholesterol/HDL-cholesterol ratio, use of lipid-modifying medication, use of anti-hypertensive medication and office systolic BP.

*$p<0.05$, **$p<0.01$, ***$p<0.001$

ESM table 3: Associations of physical activity and sedentary behavior with sVCAM-1.

	sVCAM-1		
	Model 1<br>B (95% CI)	Model 2<br>B (95% CI)	Model 3<br>B (95% CI)
Total physical activity (h/day)	-0.11 (-0.15;-0.07)***	-0.12 (-0.16;-0.08)***	-0.10 (-0.14;-0.06)***
Light intensity physical activity (h/day)	-0.08 (-0.12;-0.04)***	-0.09 (-0.13;-0.05)***	-0.07 (-0.11;-0.03)**
Moderate-to-vigorous intensity physical activity (h/day)	-0.09 (-0.13;-0.05)***	-0.10 (-0.15;-0.06)***	-0.08 (-0.12;-0.03)***
Vigorous intensity physical activity (h/day)	-0.06 (-0.10;-0.02)**	-0.07 (-0.11;-0.03)**	-0.05 (-0.09;-0.01)*
Sedentary time (h/day)	0.09 (0.05;0.14)***	0.11 (0.07;0.15)***	0.09 (0.05;0.13)***
Sedentary breaks (n/day)	-0.06 (-0.10;-0.02)**	-0.05 (-0.09;-0.01)**	-0.04 (-0.08;0.002)
Prolonged sedentary bouts (n/day)	0.10 (0.06;0.14)***	0.11 (0.07;0.15)***	0.09 (0.05;0.13)***

Regression coefficients (β) represents the increase/decrease in sVCAM-1 for every standard deviation (SD) physical activity/sedentary behavior. 1 SD total physical activity is equivalent to 0.69 h/day, 1 SD light intensity physical activity is equivalent to 1.52 h/day, 1 SD moderate-to-vigorous intensity physical activity is equivalent to 0.45 h/day, 1 SD vigorous intensity physical activity is equivalent to 0.17 h/day, 1 SD sedentary time is equivalent to

1.67 h/day, 1 SD sedentary breaks is equivalent to 8.58 breaks/day, 1 SD prolonged sedentary bouts is equivalent to 1.57 bouts/day. Model 1 was adjusted for age, sex and glucose metabolism status; model 2 was additionally adjusted for smoking, Dutch healthy diet index and level of education; model 3 was additionally adjusted for history of CVD, BMI, mobility limitation (yes/no), triacylglycerol, total cholesterol/HDL-cholesterol ratio, use of lipid-modifying medication, use of anti-hypertensive medication and office systolic BP.

*$p<0.05$, **$p<0.01$, ***$p<0.001$

ESM table 4: Associations of physical activity and sedentary behavior with sE-selectin.

	sE-Selectin		
	Model 1<br>B (95% CI)	Model 2<br>B (95% CI)	Model 3<br>B (95% CI)
Total physical activity (h/day)	-0.10 (-0.14;-0.06)***	-0.09 (-0.13;-0.05)***	-0.02 (-0.06;0.02)
Light intensity physical activity (h/day)	-0.02 (-0.06;0.02)	-0.02 (-0.06;0.02)	0.03 (-0.01;0.07)
Moderate-to-vigorous intensity physical activity (h/day)	-0.09 (-0.13;-0.05)***	-0.09 (-0.13;-0.04)***	-0.02 (-0.06;0.02)
Vigorous intensity physical activity (h/day)	-0.06 (-0.10;-0.02)**	-0.05 (-0.09;-0.02)**	-0.002 (-0.04;0.04)
Sedentary time (h/day)	0.06 (0.02;0.10)**	0.06 (0.02;0.10)**	0.01 (-0.03;0.05)
Sedentary breaks (n/day)	-0.01 (-0.05;0.03)	-0.01 (-0.05;0.03)	0.03 (-0.01;0.07)
Prolonged sedentary bouts (n/day)	0.02 (-0.02;0.06)	0.03 (-0.02;0.07)	-0.03 (-0.07;0.01)

Regression coefficients (β) represents the increase/decrease in sE-selectin behavior for every standard deviation (SD) physical activity/sedentary behavior. 1 SD total physical activity is equivalent to 0.69 h/day, 1 SD light intensity physical activity is equivalent to 1.52 h/day, 1 SD moderate-to-vigorous intensity physical activity is equivalent to 0.45 h/day, 1 SD vigorous intensity physical activity is equivalent to 0.17 h/day, 1 SD sedentary time is equivalent to 1.67 h/day, 1 SD sedentary breaks is equivalent to 8.58 breaks/day, 1 SD

prolonged sedentary bouts is equivalent to 1.57 bouts/day. Model 1 was adjusted for age, sex and glucose metabolism status; model 2 was additionally adjusted for smoking, Dutch healthy diet index and level of education; model 3 was additionally adjusted for history of CVD, BMI, mobility limitation (yes/no), triacylglycerol, total cholesterol/HDL-cholesterol ratio, use of lipid-modifying medication, use of anti-hypertensive medication and office systolic BP.

*$p<0.05$, **$p<0.01$, ***$p<0.001$

ESM table 5: Associations of physical activity and sedentary behavior with vWF.

	vWF		
	Model 1<br>B (95% CI)	Model 2<br>B (95% CI)	Model 3<br>B (95% CI)
Total physical activity (h/day)	-0.14 (-0.18;-0.10)***	-0.13 (-0.17;-0.09)***	-0.10 (-0.14;-0.06)***
Light intensity physical activity (h/day)	-0.09 (-0.14;-0.05)***	-0.09 (-0.13;-0.05)***	-0.07 (-0.12;-0.03)**
Moderate-to-vigorous intensity physical activity (h/day)	-0.12 (-0.16;-0.08)***	-0.11 (-0.15;-0.07)***	-0.08 (-0.12;-0.04)***
Vigorous intensity physical activity (h/day)	-0.06 (-0.10;-0.02)**	-0.05 (-0.10;-0.01)**	-0.03 (-0.07;0.01)
Sedentary time (h/day)	0.09 (0.05;0.13)***	0.09 (0.05;0.13)***	0.07 (0.03;0.11)**
Sedentary breaks (n/day)	-0.11 (-0.15;-0.07)***	-0.11 (-0.15;-0.07)***	-0.09 (-0.13;-0.05)***
Prolonged sedentary bouts (n/day)	0.10 (0.06;0.14)***	0.10 (0.06;0.15)***	0.08 (0.04;0.12)***

Regression coefficients (β) represents the increase/decrease in vWF for every standard deviation (SD) physical activity/sedentary behavior. 1 SD total physical activity is equivalent to 0.69 h/day, 1 SD light intensity physical activity is equivalent to 1.52 h/day, 1 SD moderate-to-vigorous intensity physical activity is equivalent to 0.45 h/day, 1 SD vigorous

intensity physical activity is equivalent to 0.17 h/day, 1 SD sedentary time is equivalent to 1.67 h/day, 1 SD sedentary breaks is equivalent to 8.58 breaks/day, 1 SD prolonged sedentary bouts is equivalent to 1.57 bouts/day Model 1 was adjusted for age, sex and glucose metabolism status; model 2 was additionally adjusted for smoking, Dutch healthy diet index and level of education; model 3 was additionally adjusted for history of CVD, BMI, mobility limitation (yes/no), triacylglycerol, total cholesterol/HDL-cholesterol ratio, use of lipid-modifying medication, use of anti-hypertensive medication and office systolic BP.

*p<0.05, **p<0.01, ***p<0.001

ESM table 6: Associations of physical activity and sedentary behavior with SAA.

	SAA		
	Model 1<br>B (95% CI)	Model 2<br>B (95% CI)	Model 3<br>B (95% CI)
Total physical activity (h/day)	-0.10 (-0.14;-0.06)***	-0.11 (-0.15;-0.07)***	-0.07 (-0.11;-0.03)**
Light intensity physical activity (h/day)	-0.07 (-0.12;-0.03)***	-0.08 (-0.12;-0.04)***	-0.06 (-0.10;-0.02)**
Moderate-to-vigorous intensity physical activity (h/day)	-0.09 (-0.13;-0.05)***	-0.10 (-0.14;-0.06)***	-0.06 (-0.10;-0.02)**
Vigorous intensity physical activity (h/day)	-0.04 (-0.08;0.000)*	-0.04 (-0.08;0.000)*	-0.01 (-0.05;0.03)
Sedentary time (h/day)	0.07 (0.02;0.11)**	0.07 (0.03;0.12)**	0.05 (0.002;0.09)*
Sedentary breaks (n/day)	-0.11 (-0.15;-0.07)***	-0.10 (-0.14;-0.06)***	-0.07 (-0.11;-0.03)***
Prolonged sedentary bouts (n/day)	0.09 (0.05;0.13)***	0.10 (0.06;0.14)***	0.06 (0.02;0.10)**

Regression coefficients (β) represents the increase/decrease SAA for every standard deviation (SD) physical activity/sedentary behavior. 1 SD total physical activity is equivalent to 0.69 h/day, 1 SD light intensity physical activity is equivalent to 1.52 h/day, 1 SD moderate-to-vigorous intensity physical activity is equivalent to 0.45 h/day, 1 SD vigorous intensity physical activity is equivalent to 0.17 h/day, 1 SD sedentary time is equivalent to 1.67 h/day, 1 SD sedentary breaks is equivalent to 8.58 breaks/day, 1 SD prolonged sedentary bouts is

equivalent to 1.57 bouts/day. Model 1 was adjusted for age, sex and glucose metabolism status; model 2 was additionally adjusted for smoking, Dutch healthy diet index and level of education; model 3 was additionally adjusted for history of CVD, BMI, mobility limitation (yes/no), triacylglycerol, total cholesterol/HDL-cholesterol ratio, use of lipid-modifying medication, use of anti-hypertensive medication and office systolic BP.

*p<0.05, **p<0.01, ***p<0.001

ESM table 7: Associations of physical activity and sedentary behavior with IL-6.

	IL-6		
	Model 1<br>B (95% CI)	Model 2<br>B (95% CI)	Model 3<br>B (95% CI)
Total physical activity (h/day)	-0.22 (-0.26;-0.18)***	-0.20 (-0.24;-0.16)***	-0.13 (-0.17;-0.09)***
Light intensity physical activity (h/day)	-0.14 (-0.18;-0.10)***	-0.13 (-0.17;-0.09)***	-0.08 (-0.12;-0.04)***
Moderate-to-vigorous intensity physical activity (h/day)	-0.21 (-0.24;-0.17)***	-0.18 (-0.22;-0.14)***	-0.12 (-0.16;-0.08)***
Vigorous intensity physical activity (h/day)	-0.14 (-0.18;-0.10)***	-0.12 (-0.16;-0.08)***	-0.07 (-0.11;-0.03)***
Sedentary time (h/day)	0.13 (0.09;0.17)***	0.12 (0.08;0.16)***	0.06 (0.03;0.10)**
Sedentary breaks (n/day)	-0.11 (-0.14;-0.07)***	-0.10 (-0.14;-0.07)***	-0.06 (-0.10;-0.02)**
Prolonged sedentary bouts (n/day)	0.14 (0.10;0.18)***	0.14 (0.10;0.18)***	0.08 (0.04;0.12)***

Regression coefficients (β) represents the increase/decrease in IL-6 for every standard deviation (SD) physical activity/sedentary behavior. 1 SD total physical activity is equivalent to 0.69 h/day, 1 SD light intensity physical activity is equivalent to 1.52 h/day, 1 SD moderate-to-vigorous intensity physical activity is equivalent to 0.45 h/day, 1 SD vigorous

intensity physical activity is equivalent to 0.17 h/day, 1 SD sedentary time is equivalent to 1.67 h/day, 1 SD sedentary breaks is equivalent to 8.58 breaks/day, 1 SD prolonged sedentary bouts is equivalent to 1.57 bouts/day. Model 1 was adjusted for age, sex and glucose metabolism status; model 2 was additionally adjusted for smoking, Dutch healthy diet index and level of education; model 3 was additionally adjusted for history of CVD, BMI, mobility limitation (yes/no), triacylglycerol, total cholesterol/HDL-cholesterol ratio, use of lipid-modifying medication, use of anti-hypertensive medication and office systolic BP.

*p<0.05, **p<0.01, ***p<0.001

ESM table 8: Associations of physical activity and sedentary behavior with IL-8.

	IL-8		
	Model 1<br>B (95% CI)	Model 2<br>B (95% CI)	Model 3<br>B (95% CI)
Total physical activity (h/day)	-0.06 (-0.10;-0.02)**	-0.04 (-0.08;0.001)	-0.01 (-0.05;0.03)
Light intensity physical activity (h/day)	-0.03 (-0.07;0.01)	-0.02 (-0.06;0.02)	0.01 (-0.04;0.05)
Moderate-to-vigorous intensity physical activity (h/day)	-0.07 (-0.11;-0.03)**	-0.05 (-0.09;-0.01)*	-0.02 (-0.06;0.02)
Vigorous intensity physical activity (h/day)	-0.04 (-0.08;-0.01)*	-0.03 (-0.07;0.01)	-0.01 (-0.05;0.03)
Sedentary time (h/day)	0.07 (0.03;0.11)***	0.06 (0.02;0.10)**	0.04 (-0.004;0.08)
Sedentary breaks (n/day)	0.02 (-0.02;0.06)	0.02 (-0.02;0.06)	0.03 (-0.004;0.07)
Prolonged sedentary bouts (n/day)	0.03 (-0.01;0.07)	0.02 (-0.02;0.06)	-0.001 (-0.04;0.04)

Regression coefficients (β) represents the increase/decrease in IL-8 for every standard deviation (SD) physical activity/sedentary behavior. 1 SD total physical activity is equivalent to 0.69 h/day, 1 SD light intensity physical activity is equivalent to 1.52 h/day, 1 SD moderate-to-vigorous intensity physical activity is equivalent to 0.45 h/day, 1 SD vigorous intensity physical activity is equivalent to 0.17 h/day, 1 SD sedentary time is equivalent to

1.67 h/day, 1 SD sedentary breaks is equivalent to 8.58 breaks/day, 1 SD prolonged sedentary bouts is equivalent to 1.57 bouts/day. Model 1 was adjusted for age, sex and glucose metabolism status; model 2 was additionally adjusted for smoking, Dutch healthy diet index and level of education; model 3 was additionally adjusted for history of CVD, BMI, mobility limitation (yes/no), triacylglycerol, total cholesterol/HDL-cholesterol ratio, use of lipid-modifying medication, use of anti-hypertensive medication and office systolic BP.

*$p<0.05$, **$p<0.01$, ***$p<0.001$

ESM table 9: Associations of physical activity and sedentary behavior with TNF-α.

	TNF-α		
	Model 1<br>B (95% CI)	Model 2<br>B (95% CI)	Model 3<br>B (95% CI)
Total physical activity (h/day)	-0.12 (-0.16;-0.08)***	-0.11 (-0.15;-0.07)***	-0.06 (-0.10;-0.02)**
Light intensity physical activity (h/day)	-0.09 (-0.13;-0.05)***	-0.09 (-0.13;-0.05)***	-0.05 (-0.09;-0.01)*
Moderate-to-vigorous intensity physical activity (h/day)	-0.12 (-0.16;-0.08)***	-0.11 (-0.16;-0.07)***	-0.06 (-0.11;-0.02)**
Vigorous intensity physical activity (h/day)	-0.10 (-0.14;-0.06)***	-0.09 (-0.13;-0.05)***	-0.06 (-0.10;-0.02)**
Sedentary time (h/day)	0.10 (0.06;0.14)***	0.10 (0.06;0.14)***	0.06 (0.01;0.10)*
Sedentary breaks (n/day)	-0.07 (-0.10;-0.03)**	-0.06 (-0.10;-0.02)**	-0.04 (-0.08;0.001)
Prolonged sedentary bouts (n/day)	0.10 (0.06;0.14)***	0.10 (0.06;0.14)***	0.06 (0.02;0.10)**

Regression coefficients (β) represents the increase/decrease in TNF-α for every standard deviation (SD) physical activity/sedentary behavior. 1 SD total physical activity is equivalent to 0.69 h/day, 1 SD light intensity physical activity is equivalent to 1.52 h/day, 1 SD moderate-to-vigorous intensity physical activity is equivalent to 0.45 h/day, 1 SD vigorous intensity physical activity is equivalent to 0.17 h/day, 1 SD sedentary time is equivalent to 1.67 h/day, 1 SD sedentary breaks is equivalent to 8.58 breaks/day, 1 SD prolonged sedentary

bouts is equivalent to 1.57 bouts/day. Model 1 was adjusted for age, sex and glucose metabolism status; model 2 was additionally adjusted for smoking, Dutch healthy diet index and level of education; model 3 was additionally adjusted for history of CVD, BMI, mobility limitation (yes/no), triacylglycerol, total cholesterol/HDL-cholesterol ratio, use of lipid-modifying medication, use of anti-hypertensive medication and office systolic BP.

*p<0.05, **p<0.01, ***p<0.001

ESM table 10: Associations of physical activity and sedentary behavior with hs-CRP.

	hs-CRP		
	Model 1<br>B (95% CI)	Model 2<br>B (95% CI)	Model 3<br>B (95% CI)
Total physical activity (h/day)	-0.20 (-0.24;-0.16)***	-0.18 (-0.22;-0.14)***	-0.10 (-0.14;-0.06)***
Light intensity physical activity (h/day)	-0.12 (-0.16;-0.08)***	-0.12 (-0.16;-0.08)***	-0.06 (-0.10;-0.03)**
Moderate-to-vigorous intensity physical activity (h/day)	-0.21 (-0.25;-0.17)***	-0.18 (-0.22;-0.14)***	-0.10 (-0.14;-0.06)***
Vigorous intensity physical activity (h/day)	-0.15 (-0.19;-0.11)***	-0.13 (-0.17;-0.09)***	-0.07 (-0.11;-0.03)***
Sedentary time (h/day)	0.12 (0.08;0.17)***	0.13 (0.08;0.17)***	0.06 (0.02;0.10)**
Sedentary breaks (n/day)	-0.12 (-0.16;-0.08)***	-0.11 (-0.15;-0.07)***	-0.06 (-0.10;-0.02)**
Prolonged sedentary bouts (n/day)	0.13 (0.09;0.17)***	0.14 (0.10;0.18)***	0.07 (0.03;0.11)***

Regression coefficients (β) represents the increase/decrease in hs-CRP for every standard deviation (SD) physical activity/sedentary behavior. 1 SD total physical activity is equivalent to 0.69 h/day, 1 SD light intensity physical activity is equivalent to 1.52 h/day, 1 SD moderate-to-vigorous intensity physical activity is equivalent to 0.45 h/day, 1 SD vigorous intensity physical activity is equivalent to 0.17 h/day, 1 SD sedentary time is equivalent to 1.67 h/day, 1 SD sedentary breaks is equivalent to 8.58 breaks/day, 1 SD prolonged sedentary

bouts is equivalent to 1.57 bouts/day. Model 1 was adjusted for age, sex and glucose metabolism status; model 2 was additionally adjusted for smoking, Dutch healthy diet index and level of education; model 3 was additionally adjusted for history of CVD, BMI, mobility limitation (yes/no), triacylglycerol, total cholesterol/HDL-cholesterol ratio, use of lipid-modifying medication, use of anti-hypertensive medication and office systolic BP.

*p<0.05, **p<0.01, ***p<0.001

ESM table 11: Associations of light intensity physical activity and sedentary behavior with low-grade inflammation, stratified by gender.

		Low-grade inflammation		
		Model 1<br>β (95% CI)	Model 2<br>β (95% CI)	Model 3<br>β (95% CI)
Men	Light intensity physical activity (h/day)	-0.10 (-0.15;-0.05)***	-0.08 (-0.13;-0.03)**	-0.03 (-0.08;0.03)
	Prolonged sedentary bouts (n/day)	0.11 (0.06;0.17)***	0.11 (0.05;0.16)***	0.04 (-0.01;0.09)
Woman	Light intensity physical activity (h/day)	-0.17 (-0.22;-0.11)***	-0.17 (-0.22;-0.11)***	-0.11 (-0.16;-0.06)***
	Prolonged sedentary bouts (n/day)	0.18 (0.13;0.24)***	0.19 (0.14;0.24)***	0.11 (0.06;0.17)***

Regression coefficients (β) represents the increase/decrease in low-grade inflammation for every standard deviation (SD) physical activity/sedentary behavior. For men: 1 SD light intensity physical activity is equivalent to 1.46 h/day, 1 SD prolonged sedentary bouts is equivalent to 1.62 bouts/day. For woman: 1 SD light intensity physical activity is equivalent to 1.44 h/day, 1 SD prolonged sedentary bouts is equivalent to 1.41 bouts/day. Model 1 was adjusted for age, sex and glucose metabolism status; model 2 was additionally adjusted for smoking, Dutch healthy diet index and level of education; model 3 was additionally adjusted for history of CVD, BMI, mobility limitation (yes/no), triacylglycerol, total cholesterol/HDL-

cholesterol ratio, use of lipid-modifying medication, use of anti-hypertensive medication and office systolic BP.

*p<0.05, **p<0.01, ***p<0.001

ESM table 12: Associations of interaction physical activity and sedentary behavior with HbA1c with biomarkers of endothelial dysfunction.

	Biomarkers of endothelial dysfunction
Total physical activity (h/day) * HbA1c	-0.004 (-0.01;0.002)
Light intensity physical activity (h/day) * HbA1c	-0.01 (-0.01;-0.003)**
Moderate-to-vigorous intensity physical activity (h/day) * HbA1c	-0.002 (-0.01;0.004)
Vigorous intensity physical activity (h/day) * HbA1c	-0.001 (-0.01;0.01)
Sedentary time (h/day) * HbA1c	0.01 (-0.001;0.01)

Regression results are presented as unstandardized coefficients (β), with 95% CI.

The analysis was adjusted for age, sex, glucose metabolism status, smoking, Dutch healthy diet index, level of education, history of CVD, BMI, mobility limitation (yes/no), triacylglycerol, total cholesterol/HDL-cholesterol ratio, use of lipid-modifying medication, use of anti-hypertensive medication and office systolic BP.

*p<0.05, **p<0.01, ***p<0.001

ESM table 13: Associations of interaction physical activity and sedentary behavior with FPG with biomarkers of endothelial dysfunction.

	Biomarkers of endothelial dysfunction
Total physical activity (h/day) * FPG	-0.05 (-0.09;-0.02)**
Light intensity physical activity (h/day) * FPG	-0.06 (-0.09;-0.03)**
Moderate-to-vigorous intensity physical activity (h/day) * FPG	-0.04 (-0.08;-0.01)*
Vigorous intensity physical activity (h/day) * FPG	-0.03 (-0.07;0.01)
Sedentary time (h/day) * FPG	0.06 (0.02;0.09)**

Regression results are presented as unstandardized coefficients (β), with 95% CI. The analysis was adjusted for age, sex, glucose metabolism status, smoking, Dutch healthy diet index, level of education, history of CVD, BMI, mobility limitation (yes/no), triacylglycerol, total cholesterol/HDL-cholesterol ratio, use of lipid-modifying medication, use of anti-hypertensive medication and office systolic BP.

*p<0.05, **p<0.01, ***p<0.001

ESM table 14: Associations of interaction physical activity and sedentary behavior with 2hPG with biomarkers of endothelial dysfunction.

	Biomarkers of endothelial dysfunction
Total physical activity (h/day) * 2hPG	-0.01 (-0.02;-0.01)**
Light intensity physical activity (h/day) * 2hPG	-0.02 (-0.02;-0.01)***
Moderate-to-vigorous intensity physical activity (h/day) * 2hPG	-0.01 (-0.02;-0.002)*
Vigorous intensity physical activity (h/day) * 2hPG	-0.01 (-0.02;0.000)
Sedentary time (h/day) * 2hPG	0.01 (0.004;0.02)**

Regression results are presented as unstandardized coefficients (β), with 95% CI. The analysis was adjusted for age, sex, glucose metabolism status, smoking, Dutch healthy diet index, level of education, history of CVD, BMI, mobility limitation (yes/no), triacylglycerol, total cholesterol/HDL-cholesterol ratio, use of lipid-modifying medication, use of anti-hypertensive medication and office systolic BP.

*p<0.05, **p<0.01, ***p<0.001

ESM table 15: Associations of physical activity and sedentary behavior with biomarkers of endothelial dysfunction.

	Biomarkers of endothelial dysfunction		
	Model 1 B (95% CI)	Model 2 B (95% CI)	Model 3 B (95% CI)
Total physical activity (h/day)	-0.16 (-0.20;-0.12)***	-0.15 (-0.19;-0.11)***	-0.09 (-0.13;-0.05)***
Light intensity physical activity (h/day)	-0.08 (-0.12;-0.04)***	-0.08 (-0.12;-0.04)***	-0.04 (-0.08;0.001)
Moderate-to-vigorous intensity physical activity (h/day)	-0.15 (-0.19;-0.11)***	-0.13 (-0.18;-0.09)***	-0.07 (-0.12;-0.03)***
Vigorous intensity physical activity (h/day)	-0.10 (-0.14;-0.06)***	-0.09 (-0.13;-0.05)***	-0.04 (-0.08;-0.004)*
Sedentary time (h/day)	0.12 (0.08;0.16)***	0.12 (0.08;0.17)***	0.08 (0.04;0.12)***
Sedentary breaks (n/day)	-0.06 (-0.10;-0.02)**	-0.06 (-0.10;-0.02)**	-0.02 (-0.06;0.02)
Prolonged sedentary bouts (n/day)	0.09 (0.05;0.14)***	0.10 (0.06;0.14)***	0.05 (0.01;0.09)*

Regression coefficients (β) represents the increase/decrease in biomarkers of endothelial dysfunction biomarker behavior for every standard deviation (SD) physical activity/sedentary behavior. 1 SD total physical activity is equivalent to 0.69 h/day, 1 SD light intensity physical activity is equivalent to 1.51 h/day, 1 SD moderate-to-vigorous intensity physical activity is

equivalent to 0.45 h/day, 1 SD vigorous intensity physical activity is equivalent to 0.17 h/day, 1 SD sedentary time is equivalent to 1.65 h/day, 1 SD sedentary breaks is equivalent to 8.61 breaks/day, 1 SD prolonged sedentary bouts is equivalent to 1.55 bouts/day. Model 1 was adjusted for age, sex and glucose metabolism status; model 2 was additionally adjusted for smoking, Dutch healthy diet index and level of education; model 3 was additionally adjusted for history of cardiovascular disease, BMI, mobility limitation (yes/no), triacylglycerol, total cholesterol/HDL-cholesterol ratio, use of lipid-modifying medication, use of anti-hypertensive medication and office systolic BP.

*p<0.05, **p<0.01, ***p<0.001

ESM table 16: Associations of physical activity and sedentary behavior with low-grade inflammation.

	Low-grade inflammation		
	Model 1<br>B (95% CI)	Model 2<br>B (95% CI)	Model 3<br>B (95% CI)
Total physical activity (h/day)	-0.19 (-0.23;-0.15)***	-0.16 (-0.20;-0.13)***	-0.08 (-0.12;-0.04)***
Light intensity physical activity (h/day)	-0.12 (-0.16;-0.08)***	-0.11 (-0.15;-0.07)***	-0.06 (-0.09;-0.02)**
Moderate-to-vigorous intensity physical activity (h/day)	-0.19 (-0.23;-0.15)***	-0.16 (-0.20;-0.12)***	-0.08 (-0.12;-0.04)***
Vigorous intensity physical activity (h/day)	-0.14 (-0.18;-0.10)***	-0.12 (-0.16;-0.08)***	-0.06 (-0.10;-0.02)**
Sedentary time (h/day)	0.14 (0.10;0.18)***	0.13 (0.09;0.17)***	0.07 (0.03;0.11)**
Sedentary breaks (n/day)	-0.09 (-0.13;-0.05)***	-0.08 (-0.12;-0.05)***	-0.03 (-0.07;0.01)
Prolonged sedentary bouts (n/day)	0.12 (0.08;0.16)***	0.12 (0.08;0.16)***	0.06 (0.02;0.09)**

Regression coefficients (β) represents the increase/decrease in low-grade inflammation for every standard deviation (SD) physical activity/sedentary behavior. 1 SD total physical activity is equivalent to 0.69 h/day, 1 SD light intensity physical activity is equivalent to 1.51 h/day, 1 SD moderate-to-vigorous intensity physical activity is equivalent to 0.45 h/day, 1 SD vigorous intensity physical activity is equivalent to 0.17 h/day, 1 SD sedentary time is

equivalent to 1.65 h/day, 1 SD sedentary breaks is equivalent to 8.61 breaks/day, 1 SD prolonged sedentary bouts is equivalent to 1.55 bouts/day. Model 1 was adjusted for age, sex and glucose metabolism status; model 2 was additionally adjusted for smoking, Dutch healthy diet index and level of education; model 3 was additionally adjusted for history of cardiovascular disease, BMI, mobility limitation (yes/no), triacylglycerol, total cholesterol/HDL-cholesterol ratio, use of lipid-modifying medication, use of anti-hypertensive medication and office systolic BP.

*p<0.05, **p<0.01, ***p<0.001

ESM table 17: Associations of physical activity and sedentary behavior with biomarkers of endothelial dysfunction.

	Biomarkers of endothelial dysfunction		
	Model 1 B (95% CI)	Model 2 B (95% CI)	Model 3 B (95% CI)
Total physical activity (h/day)	-0.18 (-0.22;-0.14)***	-0.17 (-0.21;-0.13)***	-0.10 (-0.14;-0.06)***
Light intensity physical activity (h/day)	-0.10 (-0.14;-0.06)***	-0.10 (-0.14;-0.06)***	-0.04 (-0.08;-0.003)*
Moderate-to-vigorous intensity physical activity (h/day)	-0.16 (-0.20;-0.13)***	-0.15 (-0.19;-0.11)***	-0.08 (-0.12;-0.04)***
Vigorous intensity physical activity (h/day)	-0.10 (-0.14;-0.06)***	-0.09 (-0.13;-0.05)***	-0.03 (-0.07;0.01)
Sedentary time (h/day)	0.13 (0.09;0.17)***	0.13 (0.09;0.17)***	0.07 (0.03;0.11)**
Sedentary breaks (n/day)	-0.08 (-0.12;-0.04)***	-0.08 (-0.12;-0.04)***	-0.04 (-0.07;0.001)
Prolonged sedentary bouts (n/day)	0.11 (0.07;0.15)***	0.12 (0.08;0.16)***	0.05 (0.01;0.09)**

Regression coefficients (β) represents the increase/decrease in biomarkers of endothelial dysfunciton for every standard deviation (SD) physical activity/sedentary behavior. 1 SD total physical activity is equivalent to 0.69 h/day, 1 SD light intensity physical activity is equivalent to 1.52 h/day, 1 SD moderate-to-vigorous intensity physical activity is equivalent to 0.45 h/day, 1 SD vigorous intensity physical activity is equivalent to 0.17 h/day, 1 SD

sedentary time is equivalent to 1.67 h/day, 1 SD sedentary breaks is equivalent to 8.58 breaks/day, 1 SD prolonged sedentary bouts is equivalent to 1.57 bouts/day. Model 1 was adjusted for age, sex and glucose metabolism status; model 2 was additionally adjusted for smoking, Dutch healthy diet index and level of education; model 3 was additionally adjusted for history of CVD, waist circumference, mobility limitation (yes/no), triacylglycerol, total cholesterol/HDL-cholesterol ratio, use of lipid-modifying medication, use of anti-hypertensive medication and office systolic BP.

*p<0.05, **p<0.01, ***p<0.001

ESM table 18: Associations of physical activity and sedentary behavior with low-grade inflammation.

	Low-grade inflammation		
	Model 1<br>B (95% CI)	Model 2<br>B (95% CI)	Model 3<br>B (95% CI)
Total physical activity (h/day)	-0.22 (-0.26;-0.18)***	-0.20 (-0.24;-0.16)***	-0.11 (-0.14;-0.07)***
Light intensity physical activity (h/day)	-0.14 (-0.18;-0.10)***	-0.13 (-0.17;-0.09)***	-0.06(-0.10;-0.02)**
Moderate-to-vigorous intensity physical activity (h/day)	-0.22 (-0.26;-0.18)***	-0.19 (-0.23;-0.15)***	-0.10 (-0.14;-0.06)***
Vigorous intensity physical activity (h/day)	-0.15 (-0.19;-0.11)***	-0.13 (-0.17;-0.09)***	-0.05 (-0.09;-0.02)**
Sedentary time (h/day)	0.16 (0.12;0.20)***	0.15 (0.11;0.19)***	0.07 (0.03;0.11)***
Sedentary breaks (n/day)	-0.11 (-0.15;-0.07)***	-0.10 (-0.14;-0.07)***	-0.05 (-0.09;-0.02)**
Prolonged sedentary bouts (n/day)	0.15 (0.11;0.19)***	0.15 (0.11;0.19)***	0.07 (0.03;0.11)***

Regression coefficients (β) represents the increase/decrease in low-grade inflammation for every standard deviation (SD) physical activity/sedentary behavior. 1 SD total physical activity is equivalent to 0.69 h/day, 1 SD light intensity physical activity is equivalent to 1.52 h/day, 1 SD moderate-to-vigorous intensity physical activity is equivalent to 0.45 h/day, 1 SD

vigorous intensity physical activity is equivalent to 0.17 h/day, 1 SD sedentary time is equivalent to 1.67 h/day, 1 SD sedentary breaks is equivalent to 8.58 breaks/day, 1 SD prolonged sedentary bouts is equivalent to 1.57 bouts/day. Model 1 was adjusted for age, sex and glucose metabolism status; model 2 was additionally adjusted for smoking, Dutch healthy diet index and level of education; model 3 was additionally adjusted for history of CVD, waist circumference, mobility limitation (yes/no), triacylglycerol, total cholesterol/HDL-cholesterol ratio, use of lipid-modifying medication, use of anti-hypertensive medication and office systolic BP.

*p<0.05, **p<0.01, ***p<0.001

ESM table 19: Associations of physical activity and sedentary behavior with biomarkers of endothelial dysfunction.

	Biomarkers of endothelial dysfunction		
	Model 1 B (95% CI)	Model 2 B (95% CI)	Model 3 B (95% CI)
Total physical activity (h/day)	-0.19 (-0.23;-0.14)***	-0.18 (-0.22;-0.13)***	-0.11 (-0.15;-0.07)***
Light intensity physical activity (h/day)	-0.10 (-0.15;-0.06)***	-0.10 (-0.14;-0.06)***	-0.05 (-0.10;-0.01)*
Moderate-to-vigorous intensity physical activity (h/day)	-0.16 (-0.21;-0.12)***	-0.15 (-0.19;-0.11)***	-0.09 (-0.13;-0.04)***
Vigorous intensity physical activity (h/day)	-0.10 (-0.14;-0.06)***	-0.10 (-0.14;-0.05)***	-0.04 (-0.08;-0.002)*
Sedentary time (h/day)	0.13 (0.08;0.17)***	0.13 (0.08;0.17)***	0.08 (0.03;0.12)**
Sedentary breaks (n/day)	-0.09 (-0.13;-0.05)***	-0.08 (-0.13;-0.04)***	-0.04 (-0.08;0.000)
Prolonged sedentary bouts (n/day)	0.11 (0.07;0.15)***	0.11 (0.07;0.16)***	0.06 (0.02;0.10)**

Regression coefficients (β) represents the increase/decrease in biomarkers of endothelial dysfunction for every standard deviation (SD) physical activity/sedentary behavior. 1 SD total physical activity is equivalent to 0.69 h/day, 1 SD light intensity physical activity is equivalent to 1.52 h/day, 1 SD moderate-to-vigorous intensity physical activity is equivalent to 0.45 h/day, 1 SD vigorous intensity physical activity is equivalent to 0.17 h/day, 1 SD

sedentary time is equivalent to 1.67 h/day, 1 SD sedentary breaks is equivalent to 8.58 breaks/day, 1 SD prolonged sedentary bouts is equivalent to 1.57 bouts/day. Model 1 was adjusted for age, sex, and glucose metabolism status; model 2 was additionally adjusted for smoking, Dutch healthy diet index and level of education; model 3 was additionally adjusted for history of CVD, BMI, mobility limitation (yes/no), triacylglycerol, total cholesterol/HDL-cholesterol ratio, use of lipid-modifying medication, use of anti-hypertensive medication and 24-h systolic BP.

*p<0.05, **p<0.01, ***p<0.001

ESM table 20: Associations of physical activity and sedentary behavior with low-grade inflammation.

	Low-grade inflammation		
	Model 1<br>B (95% CI)	Model 2<br>B (95% CI)	Model 3<br>B (95% CI)
Total physical activity (h/day)	-0.22 (-0.26;-0.18)***	-0.20 (-0.24;-0.16)***	-0.11 (-0.16;-0.07)***
Light intensity physical activity (h/day)	-0.14 (-0.18;-0.10)***	-0.13 (-0.17;-0.09)***	-0.07 (-0.11;-0.03)**
Moderate-to-vigorous intensity physical activity (h/day)	-0.22 (-0.26;-0.17)***	-0.19 (-0.23;-0.15)***	-0.11 (-0.15;-0.07)***
Vigorous intensity physical activity (h/day)	-0.15 (-0.19;-0.11)***	-0.13 (-0.17;-0.09)***	-0.07 (-0.11;-0.03)**
Sedentary time (h/day)	0.15 (0.11;0.19)***	0.14 (0.10;0.19)***	0.08 (0.03;0.12)***
Sedentary breaks (n/day)	-0.12 (-0.16;-0.08)***	-0.11 (-0.15;-0.07)***	-0.06 (-0.09;-0.02)**
Prolonged sedentary bouts (n/day)	0.15 (0.11;0.20)***	0.15 (0.11;0.19)***	0.08 (0.04;0.12)***

Regression coefficients (β) represents the increase/decrease in low-grade inflammation for every standard deviation (SD) physical activity/sedentary behavior. 1 SD total physical activity is equivalent to 0.69 h/day, 1 SD light intensity physical activity is equivalent to 1.52 h/day, 1 SD moderate-to-vigorous intensity physical activity is equivalent to 0.45 h/day, 1 SD

vigorous intensity physical activity is equivalent to 0.17 h/day, 1 SD sedentary time is equivalent to 1.67 h/day, 1 SD sedentary breaks is equivalent to 8.58 breaks/day, 1 SD prolonged sedentary bouts is equivalent to 1.57 bouts/day. Model 1 was adjusted for age, sex, and glucose metabolism status; model 2 was additionally adjusted for smoking, Dutch healthy diet index and level of education; model 3 was additionally adjusted for history of CVD, BMI, mobility limitation (yes/no), triacylglycerol, total cholesterol/HDL-cholesterol ratio, use of lipid-modifying medication, use of anti-hypertensive medication and 24-h systolic BP.

*$p<0.05$, **$p<0.01$, ***$p<0.001$

ESM table 21: Associations of physical activity and sedentary behavior with biomarkers of endothelial dysfunction score and low-grade inflammation score (n=2015)

	Biomarkers of endothelial dysfunction					Low-grade inflammation				
	Model 1 β (95% CI)	Model 2 β (95% CI)	Model 3 β (95% CI)	Model 4a β (95% CI)	Model 4b β (95% CI)	Model 1 β (95% CI)	Model 2 β (95% CI)	Model 3 β (95% CI)	Model 4a β (95% CI)	Model 4b β (95% CI)
Total physical activity (h/day)	-0.17 (-0.22;-0.13)***	-0.17 (-0.21;-0.13)***	-0.10 (-0.15;-0.06)***	-	-0.04 (-0.08;-0.01)*	-0.21 (-0.25;-0.16)***	-0.19 (-0.23;-0.14)***	-0.10 (-0.14;-0.06)***	-	-0.05 (-0.08;-0.01)**
Light intensity physical	-0.10 (-0.15;-0.06)***	-0.11 (-0.15;-0.06)***	-0.06 (-0.10;-0.01)**	-0.04 (-0.09;0.001)	-0.02 (-0.05;0.02)	-0.13 (-0.18;-0.09)***	-0.13 (-0.17;-0.09)***	-0.07(-0.11;-0.03)**	-0.05 (-0.09;-0.01)*	-0.04 (-0.07;-0.003)*

activity (h/day)										
Moderate-to-vigorous intensity physical activity (h/day)	-0.16 (-0.20;-0.12)***	-0.15 (-0.19;-0.11)***	-0.09 (-0.13;-0.04)***	-0.07 (-0.11;-0.02)**	-0.03 (-0.07;0.01)	-0.20 (-0.25;-0.16)***	-0.18 (-0.22;-0.14)***	-0.10 (-0.14;-0.06)***	-0.09 (-0.13;-0.04)***	-0.06 (-0.09;-0.02)**
Vigorous intensity physical activity (h/day)	-0.10 (-0.15;-0.06)***	-0.10 (-0.14;-0.05)***	-0.05 (-0.09;-0.01)*	-0.04 (-0.08;0.003)	-0.01 (-0.04;0.03)	-0.14 (-0.19;-0.10)***	-0.13 (-0.17;-0.09)***	-0.07 (-0.11;-0.03)**	-0.06 (-0.10;-0.02)**	-0.04 (-0.07;-0.01)*

Sedentary time (h/day)	0.13 (0.08;0.17)***	0.13 (0.09;0.18)***	0.08 (0.04;0.13)***	0.06 (0.01;0.10)*	0.04 (0.003;0.08)*	0.14 (0.10;0.18)***	0.14 (0.09;0.18)***	0.07 (0.03;0.11)**	0.04 (-0.01;0.08)	0.03 (-0.01;0.06)
Sedentary breaks (n/day)	-0.10 (-0.14;-0.05)***	-0.09 (-0.13;-0.05)***	-0.05 (-0.10;-0.01)**	-0.04 (-0.08;-0.002)*	-0.02 (-0.05;0.01)	-0.11 (-0.16;-0.07)***	-0.11 (-0.15;-0.07)***	-0.06 (-0.10;-0.02)**	-0.05 (-0.09;-0.01)*	-0.03 (-0.06;0.002)
Prolonged sedentary bouts (n/day)	0.11 (0.07;0.15)***	0.12 (0.07;0.16)***	0.06 (0.02;0.10)**	0.04 (-0.01;0.08)	0.03 (-0.01;0.06)	0.13 (0.09;0.17)***	0.13 (0.09;0.18)***	0.06 (0.02;0.10)**	0.03 (-0.01;0.08)	0.03 (-0.01;0.06)

Regression coefficients (β) represents the increase/decrease in biomarkers of endothelial dysfunction/low-grade inflammation for every standard deviation (SD) physical activity/sedentary behavior. 1 SD total physical activity is equivalent to 0.67 h/day, 1 SD light intensity physical activity is equivalent to 1.47 h/day, 1 SD moderate-to-vigorous intensity physical activity is equivalent to 0.43 h/day, 1 SD vigorous intensity physical

activity is equivalent to 0.16 h/day, 1 SD sedentary time is equivalent to 1.61 h/day, 1 SD sedentary breaks is equivalent to 8.29 breaks/day, 1 SD prolonged sedentary bouts is equivalent to 1.52 bouts/day. Model 1 was adjusted for age, sex and glucose metabolism status; model 2 was additionally adjusted for smoking, Dutch healthy diet index and level of education; model 3 was additionally adjusted for history of CVD, BMI, mobility limitation (yes/no), triacylglycerol, total cholesterol/HDL-cholesterol ratio, use of lipid-modifying medication, use of anti-hypertensive medication and office systolic BP. For the sedentary behavior and Light intensity physical activity, model 4a was additionally adjusted for moderate-to-vigorous intensity physical activity. For moderate-to-vigorous intensity physical activity and vigorous intensity physical activity, model 4a was additionally adjusted for sedentary time. Model 4b was additionally adjusted for low-grade inflammation (in case of endothelial dysfunction) and endothelial dysfunction (in case of low-grade inflammation)

*$p<0.05$, **$p<0.01$, ***$p<0.001$
